# Use and perceptions on reusable and non-reusable menstrual products in Spain: A mixed-methods study

**DOI:** 10.1371/journal.pone.0265646

**Published:** 2022-03-17

**Authors:** Laura Medina-Perucha, Tomàs López-Jiménez, Anna Sofie Holst, Constanza Jacques-Aviñó, Jordina Munrós-Feliu, Cristina Martínez-Bueno, Carme Valls-Llobet, Diana Pinzón Sanabria, Mª Mercedes Vicente-Hernández, Anna Berenguera

**Affiliations:** 1 Fundació Institut Universitari per a la recerca a l’Atenció Primària de Salut Jordi Gol i Gurina (IDIAPJGol), Barcelona, Spain; 2 Universitat Autònoma de Barcelona, Bellaterra (Cerdanyola del Vallès), Spain; 3 Universitat Pompeu Fabra, Barcelona, Spain; 4 Division of Country Health Policies and Systems, WHO/Europe, Copenhagen, Denmark; 5 Atenció a la Salut Sexual I Reproductiva (ASSIR) Muntanya/La Mina, Institut Català de la Salut, Barcelona, Spain; 6 Servei d’Atenció a la Salut Sexual i Reproductiva (ASSIR). Direcció Assistencial d’Atenció Primària. Institut Català de la Salut, Barcelona, Spain; 7 Sexual and Reproductive Health Care Research Group (GRASSIR), Barcelona, Spain; 8 Centro de Análisis y Programas Sanitarios (CAPS), Barcelona, Spain; 9 SomiArte Taller, Barcelona, Spain; 10 Departament d’Infermeria, Universitat de Girona, Girona, Spain; Xiamen University - Malaysia Campus: Xiamen University - Malaysia, MALAYSIA

## Abstract

**Background:**

Menstrual products are necessary goods for women and people who menstruate to manage menstruation. Understanding the use and perceptions of menstrual products is key to promote menstrual equity and menstrual health. This study aimed at assessing the use and perceptions on menstrual products among women and people who menstruate aged 18–55 in Spain.

**Methods:**

A mixed-methods study was conducted, including a cross-sectional study (N = 22,823), and a qualitative study (N = 34).

**Results:**

Participants used a combination of products. Non-reusable products were the most used, while over half used reusable products. Usage changed when data were stratified by age, gender identification, completed education, country of birth and experiencing financial issues. It also varied between trans and cis participants. Menstrual products’ use also shifted based on experiences of menstrual poverty and access to information and products. Overall, reusable products were perceived to be more acceptable than non-reusable. Barriers to use the menstrual cup were also identified, including experiences of menstrual inequity (e.g., menstrual poverty, lack of access to information or menstrual management facilities).

**Conclusion:**

Perceptions and choices of menstrual products need to be acknowledged, especially when designing and implementing menstrual policies to address menstrual inequity and menstrual health.

## Introduction

There has been a growing attention towards menstruation and menstrual management in the last few years. This mostly comes as a reaction to the lack of strategies to ensure adequate menstrual management and reduce menstrual inequities and promote menstrual health, along with a lack of research and sociopolitical awareness [1–3]. As other researchers and organisations, we advocate for menstrual health as a public health and human rights issue [4]. Menstrual products are often referred to as “feminine products”, “sanitary products” or “feminine hygiene”, all terms reinforcing menstrual-related stigma and taboo [5] and underlining menstruation as being socially expected to be concealed and managed in private spheres [6].

Non-reusable products (e.g., tampons or pads) are the most accessible, and often most conventional to use. Interestingly, nearly all research has focused on investigating the use and acceptability of reusable products (e.g., menstrual cup, reusable pads or menstrual underwear) rather than non-reusable products. The reusable products are increasingly gaining attention as new tools for menstrual management [7]. Available evidence suggests that the menstrual cup is generally acceptable [7–10] and may positively contribute to reducing menstrual-related school absenteeism [11] and thus play a part in attaining menstrual equity. Menstrual cups are also perceived to be more environmentally friendly and have less economic impact [11], although reusable products are not affordable [12] or acceptable to all women and people who menstruate (PWM). In fact, investigating the use and perceptions of menstrual products is imperative to menstrual poverty research. Based on our research, we defined menstrual poverty as: 1) not being able to afford menstrual products, 2) not being able to choose preferred products, and 3) having to prioritise menstrual products over other products or activities [13].

While most research on use and acceptability of reusable products has been conducted in African and Asian countries, studies in the Global North are mostly limited to the United States [7]. Also, research on use and acceptability of products should consider how women and PWM access and use them based on different social axes such as age or place of birth. Therefore, this study aimed at assessing the use of and perceptions on menstrual products among women and PWM aged 18–55 in Spain.

## Materials and methods

We conducted a mixed-methods study, part of the “Equity and Menstrual Health in Spain” project, including data from a cross-sectional and a qualitative study. The project adopts a critical and feminist perspective [14–16], questioning androcentric research and systemic sociopolitical structures that shape the experiences of women and PWM.

### Quantitative study

The quantitative study consisted of a cross-sectional study using an online survey (N = 22,823), completed by women and PWM aged 18–55 living in Spain at the time of data collection (24th of March-8th of July 2021). Main exclusion criteria were having entered menopause or not menstruating for over 12 consecutive months (except for pregnant and breastfeeding women and PWM).

Questions on menstrual health and menstrual inequity were included in the online survey. This was developed by the research team based on previous evidence and questionnaire design guidelines [17], along with the experience gained from the qualitative data collection. The questionnaire was piloted prior to data collection and data were collected through the Lime Survey platform (https://www.limesurvey.org). Given that response options for some survey questions were not mutually exclusive, the sum of some percentages are below or above 100%. Participants were recruited using several strategies: social media (Instagram, Twitter and WhatsApp), key persons and organisations (e.g., sexual and reproductive health centres (ASSIRs), primary healthcare centres, non-governmental organisations, other local organisations, and snowball sampling techniques. Face-to-face recruitment was done in a service for sex workers and a food bank to recruit women and PWM with limited access to information and communication technologies (N = 78).

Sample size power calculations were done for the overall quantitative study for the “Equity and Menstrual Health in Spain” project. Given the lack of research on menstrual inequity, we used a menstrual hygiene management variable from previous studies as a main variable for sample size calculations. Maximum indetermination of the main variable (proportion of 50%) was assumed. These assumptions were in order to obtain a precision of 2.5% in the confidence intervals. These estimates have been calculated assuming an alfa risk of 5%. PASS software was used for the sample size calculations [PASS 15 Power Analysis and Sample Size Software (2017). NCSS, LLC. Kaysville, Utah, USA]. A minimum of 1,535 participants were required for the quantitative study.

Descriptive statistics were performed for each variable to identify asymmetric distributions. Age was analysed as mean (SD) based on the normality of the distribution, and categorical variables were described as percentages. Analyses were performed by all menstrual products and then grouping menstrual products in non-reusable (tampons, single-use pads, panty liners) and reusable (reusable pads, menstrual cup, menstrual panties, menstrual sponge). Data analyses were stratified according to age (18–25; 26–35; 36–45; 46–55), gender identification (women, non-binary/other), self-identified as trans (yes, I don’t know, no), place of birth (Spain, Latin America, European, other), completed education (primary, secondary, university education), and financial problems in the last 12 months (always/many times, some/a few times, never). The Chi-square test was used to assess differences between socioeconomic variables by menstrual products. Stata 17.0 and SPSS 25.0 software were used for quantitative data analyses.

### Qualitative study

The qualitative study consisted of photo-elicitation semi-structured interviews (N = 34) with women and PWM (18–47 years old). Main inclusion criteria were: 1) to menstruate regularly, 3) to be 18–55 years old, and 3) live in Barcelona or surrounding areas. Not menstruating for 12 or more consecutive months (except for pregnant and breastfeeding women and PWM), or fulfilling criteria for menopause were the main exclusion criteria.

A topic guide was devised for the qualitative study (S1 Table). Two photographs were used for the photo-elicitation, although data from the photo-elicitation are not included in this manuscript. Sampling was purposive and independent from the quantitative study. Participants were recruited through social media (Instagram, Twitter and WhatsApp), key agents and organisations (e.g. ASSIRs), primary healthcare centres, non-governmental organisations, and other local organisations. Snowball sampling techniques were also applied. Efforts were directed to recruit hard-to-reach and vulnerable populations, such as people living in socio-economic deprived areas, participants from the Roma community and migrant populations. Discourse diversity in the qualitative study was ensured by recruiting participants with different characteristics (age, socio-economic context, country of origin, migrant status, cultural background, and gender identity).

Qualitative data were collected between December 2020 and February 2021 in ASSIRs, public spaces or by telephone, to comply with COVID-19 measures and participants’ preferences and availability. Data were analysed based on reflexive inductive thematic analysis [18, 19]. Only the theme on “perceptions of menstrual products” is included in this publication. The rigor and quality of the study was assessed using the Guba & Lincoln criteria [20] and the Critical Appraisal Skills Programme (CASP) tool [21].

### Ethical considerations

The study was conducted according to the guidelines of the Declaration of Helsinki, and approved by the Ethics Committee of IDIAPJGol (21st Nov 2020, Ref 19/178-P). Informed written consent was obtained from all participants involved in the study.

## Results

### Participant characteristics

A total of 22,823 women and PWM participated in the quantitative study. Mean age was 33.2 (*SD =* 8.7), ranging between 18 and 55. Participants mostly identified as women (96.8%). Non-binary participants represented the 1.4% of the sample, and 0.8% identified as trans. Almost all participants were born in Spain (93.4%) and had Spanish nationality (96.1%). Almost half were employed full-time (47.5%) and more than half had completed university-level education (69.4%). Almost half had had financial problems in the last year (43.7%) (see Table 1).

**Table 1:** Sociodemographic characteristics quantitative study (N = 22,823).

Variable	N (22,823)	%
Age (18–55)	*Mean =* 33.2 (*SD =* 8.7)	
Gender identification		
Women	22,100	96.8%
Non-binary/Other	723	3.2%
Trans		
Yes	175	0.8%
Don’t know	155	0.7%
Place of birth		
Spain	20,943	93.4%
Latin America	841	3.8%
Europe	501	2.2%
Other	126	0.6%
Administrative situation		
Spanish Nationality	21,785	96.1%
Permanent Residency	678	3.0%
Temporary Residency	172	0.8%
No permit/being processed	45	0.2%
Employment situation		
Employed full-time	10,834	47.5%
Employed part-time	3,914	17.1%
Studying full-time	3,896	17.1%
Self-employed	2,050	9.0%
Studying part-time	1,934	8.5%
Unemployment benefits/COVID-19 benefits	1,831	8.0%
Homemaking/caregiver	1,134	5.0%
Receiving benefits/Retired	163	0.7%
Completed education		
University studies	15,811	69.4%
Secondary education	6,728	29.5%
Primary education	217	1.0%
No formal education completed	35	0.2%
Caregiving for someone else		
Yes	7,518	33.1%
Financial problems in the last year		
Always/many times	2,707	12.1%
Sometimes/a few times	7,056	31.6%
Never	12,582	56.3%

SD: Standard Deviation.

Participants in the qualitative study were 34 women (N = 31) and PWM (N = 3) between 18 and 47 years old. Most were born in Spain (N = 30) and had Spanish nationality (N = 30). Over half were working at the time of the interviews (N = 19) and had completed university studies (N = 19). Thirteen had had financial issues in the 12 months preceding data collection (see Table 2).

**Table 2:** Participant characteristics qualitative study (N = 34).

ID	Age	Birthplace	Administrative status	Employment status	Economic issues in the last year	Completed education	Gender identity	Trans	Interview location
P1	27	Spain	Spanish nationality	No employment/income	Yes, sometimes	Primary education	Woman	No	ASSIR
P2	40	Spain	Spanish nationality	Works full-time	No	Secondary education	Woman	No	ASSIR
P3	23	Spain	Spanish nationality	Maternity leave	Yes, sometimes	Professional education	Woman	No	ASSIR
P4	24	Spain	Spanish nationality	Works full-time	No	University studies	Woman	No	Telephone
P5	25	Spain	Spanish nationality	Works full-time	No	University studies	Woman	No	Telephone
P6	29	Spain	Spanish nationality	Self-employed	No	University studies	Not sure	Not sure	Telephone
P7	33	Spain	Spanish nationality	Works full-time	No	University studies	Woman	No	Telephone
P8	35	Spain	Spanish nationality	Works full-time	No	University studies	Woman	No	Telephone
P9	24	Spain	Spanish nationality	Works full-time	Yes, always	University studies	Woman	No	Telephone
P10	33	Spain	Spanish nationality	Works full-time	No	University studies	Woman	No	Telephone
P11	33	Spain	Spanish nationality	Works full-time	No	University studies	Woman	No	Telephone
P12	25	Spain	Spanish nationality	Work full-time; Studies part-time	No	University studies	Woman	No	Public space
P13	25	Spain	Spanish nationality	Works full-time	No	University studies	Woman	No	Public space
P14	26	Spain	Spanish nationality	Studies full-time	No	University studies	Woman	No	Public space
P15	25	Spain	Spanish nationality	Works part-time	No	University studies	Woman	No	Telephone
P16	47	Spain	Spanish nationality	Works full-time	No	Professional education	Woman	No	Telephone
P17	34	Spain	Spanish nationality	Works full-time	No	University education	Woman	No	Telephone
P18	23	Spain	Spanish nationality	Medical leave; Studies part-time	Yes, sometimes	Professional education	Woman and non-binary	Not sure	Telephone
P19	25	Spain	Spanish nationality	Works part time; Studies full-time	Yes, sometimes	Secondary education	Woman	No	Telephone
P20	20	Spain	Spanish nationality	Studies full-time	Not sure	Secondary education	Woman	No	Telephone
P21	35	Spain	Spanish nationality	Self-employed; Studies part-time;	No	University studies	Woman	No	Telephone
P22	18	Spain	Spanish nationality	Studies full-time	Yes, sometimes	Secondary education	Woman	No	Public space
P23	28	Spain	Spanish nationality	Works full-time	No	University education	Woman	No	Telephone
P24	20	Spain	Spanish nationality	Studies full-time	No	Secondary education	Non-binary	Yes	Telephone
P25	37	Morocco	Permanent residence	Works full-time	No	Professional education	Woman	No	Telephone
P26	24	Spain	Spanish nationality	Works part-time	Yes, sometimes	Professional education	Woman	No	Telephone
P27	35	Colombia	Spanish nationality	Unemployed	Yes, sometimes	University studies	Woman	No	Telephone
P28	37	Spain	Spanish nationality	Works full-time	No	University studies	Woman	No	Telephone
P29	23	Argentina	Refugee status	No income	Yes, sometimes	Secondary education	Woman	No	ASSIR
P30	22	Spain	Permanent residence	Works full-time	Yes, sometimes	Professional education	Woman	No	ASSIR
P31	25	Pakistan	Permanent residence	Works full-time	Yes, sometimes	Professional education	Woman	No	Public space
P32	29	Spain	Spanish residence	Works full time	No	University studies	Woman	No	Telephone
P33	28	Spain	Spanish nationality	Works full time	Yes, sometimes	University studies	Woman	No	Telephone
P34	38	Brazil	Spanish nationality	Unemployed	Yes, sometimes	Professional education	Woman	No	ASSIR

*ASSIR = sexual and reproductive healthcare centre.

### Use of menstrual products: Quantitative analyses

Participants (N = 22,823) used a combination of menstrual products to manage menstruation (see Tables 3 and 4). Overall, while 69.7% used non-reusable menstrual products, 54.9% used reusable menstrual products. Single-use menstrual pads were the most used product (60.6%), followed by panty liners (49.7%), the menstrual cup (48.4%), tampons (42.6%), reusable pads (15%), menstrual panties (8.7%), and menstrual sponges (0.7%). Products not designed for menstruation were also used; 5.6% reported toilet paper use, 1.4% used more than one pair of underwear to retain menstrual blood, and 0.5% used diapers. A small but compelling number of women and PWM (3.7%) did not use any product for menstrual retention (free bleeding).

**Table 3:** Use of non-reusable and reusable menstrual products by age, gender identity, trans identification, country of birth, completed education and financial constraints (last 12 months) (N = 22,823).

	Non-reusable menstrual products			Reusable menstrual products		
	Yes (N(%))	No (N(%))	Pvalue	Yes (N(%))	No (N(%))	Pvalue
Age						
18–25	3378 (73.1)	1388 (26.9)	<0.001	2617 (50.7)	2549 (49.3)	<0.001
26–35	4763 (62.3)	2877 (37.7)		4899 (64.1)	2741 (35.9)	
36–45	4922 (71.6)	1957 (28.4)		3692 (53.7)	3187 (46.3)	
46–55	1584 (82.9)	326 (17.1)		646 (33.8)	1264 (66.2)	
Gender identity						
Woman	14583 (69.7)	6331 (30.3)	0.373	11498 (55.0)	9416 (45.0)	0.163
Non-binary/Other	464 (68.1)	217 (31.9)		356 (52.3)	325 (47.7)	
Trans						
Yes	121 (69.9)	52 (30.1)	0.888	76 (43.9)	97 (56.1)	0.006
I don’t know	97 (67.8)	46 (32.2)		71 (49.7)	72 (50.3)	
No	14829 (69.7)	6450 (30.3)		11707 (55.0)	9572 (45.0)	
Place of birth						
Spain	13787 (69.5)	6039 (30.5)	0.024	10944 (55.2)	8882 (44.8%)	0.001
Latin America	543 (69.0)	244 (31.0)		420 (53.4)	367 (46.6)	
Europe	335 (70.4)	141 (29.6)		261 (54.8)	215 (45.2)	
Other	98 (82.4)	21 (17.6)		44 (37.0)	75 (63.0)	
Completed education						
Primary education	190 (84.1)	36 (15.9)	<0.001	63 (27.9)	163 (72.1%)	<0.001
Secondary education	4720 (74.7)	1597 (25.3)		2991 (47.3)	3326 (52.7%)	
University education	10123 (69.7)	4903 (32.6)		8792 (58.5)	6234 (41.5%)	
Financial constraints (<12 months)						
Always/Many times	1747 (66.8)	801 (31.4)		1417 (55.6)	1131 (44.4)	0.876
Some/A few times	4612 (69.3)	2041 (30.7)	0.532	3680 (55.3)	2973 (44.7)	
Never	8318 (69.7)	3621 (30.3)		6577 (55.1)	5362 (44.9)	

*Note*. The use of reusable and non-reusable products were not mutually exclusive.

**Table 4:** Use of menstrual products by age, gender identity, trans identification, country of birth, completed education and financial constraints (last 12 months) (N = 22,823).

	Tampons	Single-use pads	Reusable pads	Panty liners	Menstrual cup	Menstrual panties	Menstrual sponges	>1 regular panties	Nappies	Toilet paper	Free bleeding
	N (%)	N (%)	N (%)	N (%)	N (%)	N (%)	N (%)	N (%)	N (%)	N (%)	N (%)
**Age**											
18–25	2217 (42.9)‡	3436 (66.59)‡	503 (9.7)‡	2540 (49.2)‡	2429 (47.0)‡	201 (3.9)‡	16 (0.3)*	85 (1.6)	26 (0.5)*	312 (6.0)‡	145 (2.8)‡
26–35	2931 (38.4)	3955 (51.8)	1462 (19.1)	3452 (45.2)	4343 (56.8)	866 (11.3)	57 (0.7)	91 (1.2)	31 (0.4)	522 (6.8)	372 (4.9)
36–45	3025 (44.0)	4254 (61.8)	1112 (16.2)	3595 (52.3)	3136 (45.6)	734 (10.7)	60 (0.9)	97 (1.4)	27 (0.4)	297 (4.3)	426 (3.6)
46–55	1024 (53.6)	1443 (75.5)	160 (8.4)	1147 (60.1)	544 (28.5)	79 (4.1)	17 (0.9)	21 (1.1)	19 (1.0)	85 (4.5)	34 (1.8)
**Gender**											
Woman	8983 (43.0)‡	12678 (60.6)	3109 (14.9)*	10485 (50.1)‡	10156 (48.6)*	1816 (8.7)	142 (0.7)	275 (1.3)*	93 (0.4)‡	1162 (5.6)*	735 (3.5)‡
Non-binary/Other	214 (31.4)	410 (60.2)	128 (18.8)	249 (36.6)	296 (43.5)	64 (9.4)	8 (1.2)	19 (2.8)	10 (1.5)	54 (7.9)	62 (9.1)
**Trans**											
Yes	40 (23.1)‡	113 (65.3)	29 (16.8)	69 (39.9)‡	63 (36.4)‡	12 (6.9)	4 (2.3)‡	5 (2.9)‡	3 (1.7)*	20 (11.6)*	14 (8.1)‡
I don’t know	43 (30.1)	84 (58.7)	21 (14.7)	49 (34.3)	55 (38.5)	13 (9.1)	4 (2.8)	7 (4.9)	2 (1.4)	8 (5.6)	14 (9.8)
No	9114 (42.8)	12891(60.6)	3187 (15.0)	10616 (49.9)	10334 (48.6)	1855 (8.7)	142 (0.7)	282 (1.3)	98 (0.5)	1188 (5.6)	769 (3.6)
**Place of birth**											
Spain	8503 (42.9)‡	11988 (60.5)*	2993 (15.1)	10020 (50.5)‡	9658 (48.7)*	1738 (8.8)	143 (0.7)	271 (1.4)	96 (0.5)	1099 (5.5)*	726 (3.7)
Latinamerica	270 (34.3)	482 (61.2)	105 (13.3)	278 (35.3)	374 (47.5)	61 (7.8)	3 (0.4)	8 (1.0)	4 (0.5)	62 (7.9)	30 (3.8)
Europe	203 (42.6)	285 (59.9)	78 (16.4)	192 (40.3)	220 (46.2)	46 (9.7)	4 (0.8)	9 (1.9)	2 (0.4)	32 (6.7)	19 (4.0)
Other	46 (38.7)	93 (78.2)	10 (8.4)	50 (42.0)	36 (30.3)	8 (6.7)	0 (0.0)	3 (2.5)	0 (0.0)	6 (5.0)	7 (5.9)
**Completed education**											
Primary education	97 (42.9)*	167 (73.9)‡	15 (6.6)‡	98 (43.4)	54 (23.9)‡	6 (2.7)‡	0 (0.0)	1 (0.4)	5 (2.2)‡	14 (6.2)	5 (2.2)
Secondary education	2784 (44.1)	4176 (66.1)	707 (11.2)	3109 (49.2)	2670 (42.3)	321 (5.1)	35 (0.6)	97 (1.5)	39 (0.6)	390 (6.2)	219 (3.5)
University education	6306 (42.0)	8734 (58.1)	2511 (16.7)	7519 (50.0)	7721 (51.4)	1552 (10.3)	114 (0.8)	194 (1.3)	59 (0.4)	810 (5.4)	570 (3.8)
**Financial problems (<12 months)**											
Always/Many times	928 (36.4)‡	1534 (60.2)	451 (17.7)‡	1033 (40.5)‡	1218(47.8)	200 (7.8)*	24 (0.9)*	52 (2.0)*	23 (0.9)*	222 (8.7)‡	170 (6.7)‡
Some/A few times	2709 (40.7)	4028 (60.5)	1049 (15.8)	3100 (46.6)	3245 (48.8)	544 (8.2)	56 (0.8)	93 (1.4)	29 (0.4)	422 (6.3)	293 (4.4)
Never	5337 (44.7)	7191 (60.2)	1698 (14.2)	6359 (53.3)	5825(48.8)	1121 (9.4)	70 (0.6)	141 (1.2)	44 (0.4)	541 (4.5)	323 (2.7)

‡P-value<0.001.

*P-value<0.05. If no label is specified, then p-value>0.05. *Note*. The use of different menstrual products was not mutually exclusive.

There were some significant differences in menstrual products’ use when data were stratified by age, gender identity, trans identification, country of birth, completed education and financial constraints in the last 12 months (see Tables 3 and 4). Significant differences were found in the usage of menstrual products by age groups. Participants aged 26–35 were the ones using more reusable products (64.1%), and particularly the menstrual cup (56.8%), reusable pads (19.1%) and menstrual panties (11.3%). The eldest group used non-reusable products the most (82.9%), including the use of tampons (53.6%) and single-use pads (75.5%).

No significant differences were found in the use of non-reusable and reusable products by gender identification. However, similarly to trans people, non-binary participants used tampons less frequently (31.4%), compared to participants identifying as women (43%). Instead, they used reusable pads more commonly (18.8%) than women (14.9%). The menstrual cup was used more by participants who identified as women (48.6%), compared to non-binary people (43.5%). Instead, non-binary participants used reusable pads (18.8%) more than those identifying as women (14.9%). Significant differences were also found between non-binary people and women in the use of panty liners (36.6% vs 50.1%), the use of more than one panty for menstrual management (2.8% vs 1.3%), nappies (1.5% vs 0.4%), toilet paper (7.9% vs 5.6%) and free bleeding (9.1% vs 3.5%).

Trans people used significantly less reusable products (43.9%) compared to cis women (55%). Compared to people who did not identify as trans, trans people used significantly less tampons (42.8% vs 23.1%) and the menstrual cup (48.6% vs 36.4%). It was also more common for trans people to use toilet paper (11.6%) or no menstrual products (8.1%), compared to cis women (5.6% and 3.6% respectively).

When stratifying findings by place of birth, participants born in Spain (69.5%), Latin American (69.0%), and European countries (70.4%) used significantly less non-reusable products, compared to participants born in other countries (82.4%). Participants born in Spain, Latin America or European countries used significantly more reusable products, compared to those born in other countries (55.2%, 53.4%, 54.8%, 37.0% respectively).

Differences in the use of non-reusable and reusable products significantly differed depending on participants’ completed education. Those who had finalised primary education used significantly more non-reusable products (84.1%), including the use of single-use pads (73.9%). Participants who had accessed university studies used significantly more reusable products (58.5%) and particularly reusable pads (16.7%), the menstrual cup (51.4%) and menstrual panties (10.3%).

Interestingly, no significant differences were found in the usage of non-reusable and reusable products between participants who had experienced financial constraints in the last 12 months, and those who did not. Significant differences were only found when looking at the use of specific products. Using reusable pads (17.7%), toilet paper (8.7%), more than one pair of underwear (2.0%), not using any menstrual products (6.7%), and diapers (0.9%) was more common among participants who experienced financial problems (<12 months) always/many times.

### Perspectives on menstrual products: Qualitative analyses

Drawing from the qualitative data (N = 34), participants used a variety of menstrual products: single-use products like pads, tampons, and panty liners and reusable products like menstrual cups, cloth pads and menstrual panties. Others would sometimes use toilet paper when menstrual products were not available:*“Well*, *what did I do*? *A lot of* (toilet) *paper*. *Until I could get one* (menstrual product) *to change*, *of course*, *because a pad (…)*, *it’s more comfortable than paper*. *But I’m not going to fool you*, *sometimes I didn’t have any*, *so* (I used) *paper”- P3*. One woman (P25), who was born and raised in Morocco, explained that she used cotton cloths when she was younger. She used to throw them away after using them as she was disgusted by having to wash blood stains. Free bleeding was also mentioned by a few participants; it was mainly practiced at home and on low-bleeding days. A few participants chose menstrual products based on their perceptions of them influencing their menstrual flow or pain. For instance, tampons and the menstrual cup were associated with feeling more pain as they had to be inserted in the vagina (P7).

When it came to single-use products, branded products were generally perceived as being the “good products”, where non-branded products were seen as the “bad ones” even though some found their favourite products among the latter. Some participants reported irritations from non-reusable products, especially from non-branded tampons, and shared their concerns on the toxic shock syndrome caused by tampons: *“I recommend reusable products to everyone I know*. *Because they last longer*, *they are cheaper in the long-term and you do not have the “toxic shock syndrome” or* (the risk of) *them causing irritations or that the chemicals they have led to an infection”-P20*. Interestingly, this may suggest that reusable products are perceived as a way to avoid the risk of toxic shock syndrome.

Tampons were thought to be practical but mostly disliked for being uncomfortable, causing irritations and the risk of the toxic shock syndrome. A few participants (P3, P19, P22, P25) shared their reluctance or barriers to use tampons or other products based on that they had to be inserted in the vagina. These participants were from the Roma community, migrants or second-generation migrants. These findings open an interesting line of investigation on the influence of sociocultural background in menstrual products’ perceptions: *“It creeped me out a bit also with tampons*, *I took some time to dare try it*. *Also because they are not used in Jordan”-P19*.

Pads were generally perceived as being traditional, the *“least bad”—P26*, often used at night and in combination with other products. They were thought to be the most absorbent products, so they were *“safer to not stain in public places”- P31*. They were also perceived negatively when they could be seen through clothes: *“The good thing about them* (specific brand of pads) *is that they are super thin*, *so you go with jeans or tights*, *and … (…)*. *Nothing is seen”–P3*. Non-reusable “plastic-like” pads were avoided by one participant (P22) as they did not transpire.

As for reusable menstrual products, the menstrual cup was the most popular, mainly among participants with university studies. It was perceived as cleaner, comfortable, environmentally-friendly, cheaper (in the long-term), allowed to learn about one’s menstruation and body, and was healthier for one’s body. Other reasons for using the cup were that it had to be changed less often, and did not smell bad (like non-reusable products). Barriers to use it mentioned were vaginal/vulvar pain after using it for a few days, finding adequate menstrual management spaces, boiling it to sterilise it when sharing a home (“*it is disgusting for other people”-P14*, *P15*, *P28*), and perceptions that it may increase menstrual bleeding (P16), may come out from the vagina while exercising (P22), or that it does not allow menstrual blood to flow out of the vagina (P6). The menstrual cup was usually recommended by friends and was perceived to be for “*younger people”* (P15) or “hippies” (P2). Similarly, to the menstrual cup, menstrual panties and reusable pads were perceived as expensive but comfortable to use. They were mainly used in combination with the menstrual cup.

## Discussion

This study aimed at assessing the use of and perceptions on menstrual products among women and PWM aged 18–55 in Spain. Findings from this study indicate that women and PWM in Spain use a combination of menstrual products. Non-reusable menstrual products were the most used, although more than half used reusable products, and especially the menstrual cup. Usage of menstrual products significantly differed based on age, gender identification, identifying as trans, place of birth, completed education, country of birth and experiencing financial issues. Perceptions and barriers to using menstrual products, along with barriers for menstrual management, were identified. Qualitative data have deepened and strengthened the quantitative findings, providing insights on the personal and structural reasons behind the use of different menstrual products. Perceptions on menstrual products, related fears and structural barriers for menstrual management, especially in public spaces, were identified in qualitative data. The findings support that menstrual inequity needs to be acknowledged as related to menstrual products’ use [13].

Findings by age groups indicated that younger women and PWM use reusable menstrual products more, compared to older participants. Particularly those between 26 and 35 were the ones reporting the highest use of reusable products. An explanation for these differences may be the differential access to menstrual learnings and opportunities to access a variety of menstrual products across generations [7, 22, 23]. Besides, the fact that participants from the youngest age group reported less use of reusable products than older participants may be related to agency and time for self-learnings.

Although sample sizes were small for some categories, the use of reusable products was higher in participants born in Latin America, Spain and other European countries, compared to participants born in other countries. Supported by the qualitative findings, this suggests that use and perceptions on menstrual products vary depending on the socio-cultural context. Therefore, socio-cultural and contextual factors need to be accounted for to understand the use and acceptability of menstrual products [12, 24]. For instance, some participants in our study appeared to be uncomfortable about the idea of using menstrual products that had to be inserted in the vagina. However, other factors such as age and education may intersect with place of birth and may provide a more comprehensive perspective on the differential usage of products. Future studies could explore how women and PWM may adopt (or not) public discourses and preferences on menstrual products. Research involving migrant women and PWM could be particularly meaningful.

Trans people used more non-reusable than reusable products. The use of toilet paper and not using products to manage menstruation was significantly more common among trans people and non-binary people, compared to cis participants and those identifying their gender as women. Acknowledging the needs and preferences of trans, intersex and non-binary people who menstruate is crucial [25, 26]. Besides, distress among non-binary, intersex and trans people who menstruate resulting from packaging and advertising of menstrual products portraying menstruation as to represent “hegemonic femininity”, and the fact that many menstrual products need to be inserted in the vagina has already been discussed [27–29]. The latter can also be inferred from our results.

The relationship between menstrual poverty and the use of menstrual products also needs to be discussed. Findings from the “Equity and Menstrual Health in Spain” study already indicate that menstrual poverty affects 22.2–39.9% of adult women and PWM in Spain [Medina-Perucha et al., *in prep*]. Data presented in this article suggest that experiencing financial issues may shape decisions made in relation to menstrual products’ use, by using more reusable pads, toilet paper, the use of more than one regular panty to contain menstrual blood, the use of no menstrual products, and diapers for menstrual management. Furthermore, toilet paper was reported to be used when menstrual products were not available in the qualitative study. However, the practice of free bleeding should be interpreted with caution, as the qualitative data indicated that some women and PWM may limit free bleeding when being at home and not bleeding abundantly. Thus, we cannot infer that not using menstrual products may always be associated with a lack of access to menstrual products. Instead, free bleeding is an active choice for some women and PWM, which could have also increased during COVID19 especially among people working from home.

Furthermore, menstrual inequity needs to be discussed when referring to the use and perceptions of menstrual products. Informed choice on menstrual products has been argued to be limited, partly due to commercial interests and the unbalanced information available on different menstrual products [7, 22, 30]. As Tarzibachi argues [31], menstrual taboo and stigma were reinforced by publicists as menstrual products were marketed as required for “feminine protection” and related to “hygiene” in the 1920s-1950s. Once women in high income countries started to enter the male-dominated workforce, menstrual products were framed as for “women’s liberation”, relegating menstruation even further to “intimate spheres”. We can also argue that the role of health professionals in offering informed-based advice for different menstrual products is underexplored. Instead, choices on menstrual products are often based on friends’ recommendations or self-learnings. Although community learnings and networks for menstrual management should be promoted, training to health professionals is necessary so that healthcare services can better respond to menstrual-related consultations [13]. In our study, usage of reusable products was also more common among participants who had completed university education, compared to those who did not. This suggests that the access to products such as the menstrual cup may not be equal to all. Instead, those who may have more access to menstrual learnings and have a more privileged social situation may have a wider access to choose how to manage their menstruation, including which products to use.

Overall, our study indicates that the menstrual cup is an acceptable product for menstrual management. Participants thought it was comfortable, cheaper in the long-term, did not smell bad (like non-reusable products do) and it allowed for women and PWM to know more about their menstruation and body. The fact that the menstrual cup is an environmentally-friendly option was another reason for participants to use it, as other research has found [32]. Previous evidence also supports our findings on the acceptability of the menstrual cups as a more comfortable and preferred product, compared to non-reusable products [8, 9].

Even if menstrual cups are more sustainable due to their durability (around 10 years), and have a reduced environmental impact as they do not use non-degradable materials [33], usage barriers need to be acknowledged. As reported by participants in our qualitative study, some women and PWM may experience pain when inserting the cup. Other barriers identified in our study were that some participants perceived that the menstrual cup could increase menstrual flow or dysmenorrhea. One participant also believed that the cup could fall off the vagina when exercising. In other research, women reported feeling a constant need to urinate, fear and/or experiencing leakage [7], and the concern that the menstrual cup could “get stuck” in the vagina [34]. Other concerns were related to the menstrual cup compromising fertility or leading to “losing virginity” [11]. Besides, despite the menstrual cup seems to be a cost-effective and acceptable option for menstrual management, menstrual education commonly fails to inform about all menstrual products available [22, 23]. Menstrual cups are not always acceptable and usually require a learning period of 2–5 months [23]. Besides, they do not always adapt to each person’s anatomy.

In our study, reusable menstrual pads were acceptable as an expensive but comfortable option to combine with other menstrual products. Reusable menstrual pads have already been positively evaluated [35], although their use might not increase social participation during menstruation [36]. A study evaluating acceptability of reusable underwear in a refugee setting in Greece showed that participants thought these products to be acceptable but not more beneficial than other conventional products and menstrual management practices. The use of menstrual underwear, in combination with other conventional products, was perceived as dignifying [37].

Taboo and stigma surrounding menstruation are also barriers for menstrual management, as apparent in our findings. Some participants explained how having to wash reusable menstrual products in shared/public spaces limited the use of the menstrual cup and reusable pads. They referred to barriers in the access to adequate menstrual management facilities, disposal of menstrual products [7, 33] and spaces to wash and dry reusable products [12] suggested in previous research. Participants’ narratives also suggested that taboo and stigma may limit the use of some non-reusable menstrual products. For instance, single-use pads may not be acceptable if they can be seen through clothes and, therefore, they are not useful to conceal menstruation. Our findings also indicate that young adult women and PWM use reusable menstrual products more. This suggests that agency among younger generations may be stronger, to actively challenge the barriers to manage menstruation in public spaces, and particularly when using products such as the menstrual cup.

On the other hand, as Grose and Grabe discussed [38], adopting androcentric views and experiencing self-objectification might lead to women and PWM to use disposable (and unhealthier) menstrual products. Interestingly, in our study, different menstrual products were perceived to be healthier than others. While the menstrual cup was perceived to be healthier, tampons were depicted as to be related to the toxic shock syndrome and causing vaginal irritations [39]. Branded products were also thought to be healthier than non-branded ones, shifting participants’ decisions on menstrual products’ use [7, 22, 30].

### Limitations

While the quantitative study included a large sample of women and PWM in Spain, the sample is not representative of the Spanish population. Besides, the sample size for some participant groups (e.g., women and PWM born in “other countries”) was small, thus findings need to be interpreted with caution. We also acknowledge the potential negative impact of having used online platforms for recruitment and data collection (for the quantitative study), limiting the participation of people affected by the digital divide. Furthermore, over half of the participants in the quantitative study had completed university education and were employed, thus limiting the representation of women and PWM with a lower educational level, access to employment and socio-economic resources. As for the qualitative study, conducting interviews in ASSIRs may have limited participants’ responses. Even if the researchers attempted to include more PWM in the qualitative study, only a few participated. In addition, women and PWM with functional diversity were not actively included in the study. Lastly and similarly to the quantitative study, the digital divide may have also affected the recruitment process and limited participation. However, this study can greatly contribute to understand how women and PWM in Spain use menstrual products and manage their menstruation.

## Conclusions

Adult women and PWM in Spain use a variety, and often a combination, of menstrual products to manage menstruation. Although non-reusable products are the most commonly used, reusable products were used by over half participants. The use of menstrual products varies depending on age, completed education, country of birth and experiencing financial issues. Usage of menstrual products also differed between trans and non-binary participants and cis women. Reusable products were most commonly used among cis women, between 26 and 35 years old, born in Latin America, Spain or other European countries, and those who had completed university education. Usage of non-reusable products was higher among women and PWM aged 46 to 55, born in non-European or Latin American countries and who had only completed primary education. Reusable products (and especially the menstrual cup) were generally perceived to be more acceptable. The menstrual cup was a comfortable and environmentally-friendly option that also promoted body literacy. Choices on menstrual products are shifted by socio-cultural factors, including experiences of menstrual poverty and the unequal and limited opportunities to access information and preferred menstrual products. Future research and policy actions should involve women and PWM in policymaking processes and research projects, and focus on addressing menstrual inequity to ensure positive and healthy menstrual management. Policies should support agency and autonomy of women and PWM in making informed-choices on menstrual products. Intersectional approaches and participatory action research considering the experiences and needs of vulnerable populations may be particularly relevant. Furthermore, research on menstrual inequity and menstrual health needs to continue and attend to the needs of women and PWM.

## Supporting information

S1 Table Interview topic guide. (DOCX)
